# Supplementary material for: Marine viruses disperse bidirectionally along the natural water cycle
Source: Nat Commun. 2023 Oct 10;14:6354. doi: 10.1038/s41467-023-42125-5 (PMC10564846; doi:10.1038/s41467-023-42125-5)
Supplement: Supplementary file 1 — Supplementary information [file 41467_2023_42125_MOESM1_ESM.pdf]

Supplementary Material
Marine viruses disperse bidirectionally along the natural water cycle

Janina Rahlff[1,2,8*], Sarah P. Esser[1,3], Julia Plewka[1,3], Mara Elena Heinrichs[4], André Soares[1,3], Claudio Scarchilli[5], Paolo Grigioni[5], Heike Wex[6], Helge-Ansgar Giebel[4,9], Alexander J. Probst[1,3,7]

[1]Group for Aquatic Microbial Ecology, University of Duisburg-Essen, Department of Chemistry, Environmental Microbiology and Biotechnology (EMB), 45141 Essen, Germany
[2]Centre for Ecology and Evolution in Microbial Model Systems (EEMiS), Department of Biology and Environmental Science, Linnaeus University, 39231 Kalmar, Sweden
[3]Environmental Metagenomics, Research Center One Health Ruhr of the University Alliance Ruhr, University of Duisburg-Essen, 45141 Essen, Germany
[4]Institute for Chemistry and Biology of the Marine Environment (ICBM), Carl von Ossietzky University of Oldenburg, 26129 Oldenburg, Germany
[5]Italian National Agency for New Technologies, Energy and Sustainable Economic Development (ENEA), 00123 Rome, Italy
[6]Atmospheric Microphysics, Leibniz Institute for Tropospheric Research (TROPOS), 04318 Leipzig, Germany
[7]Centre of Water and Environmental Research (ZWU), University of Duisburg-Essen, 45141 Essen, Germany

[8]Present address: Aero-Aquatic Virus Research Group, Faculty of Mathematics and Computer Science, Friedrich Schiller University Jena, 07743 Jena, Germany
[9]Present address: Institute for Chemistry and Biology of the Marine Environment (ICBM), Center for Marine Sensors (ZfMarS), Carl von Ossietzky University of Oldenburg, 26382 Wilhelmshaven, Germany

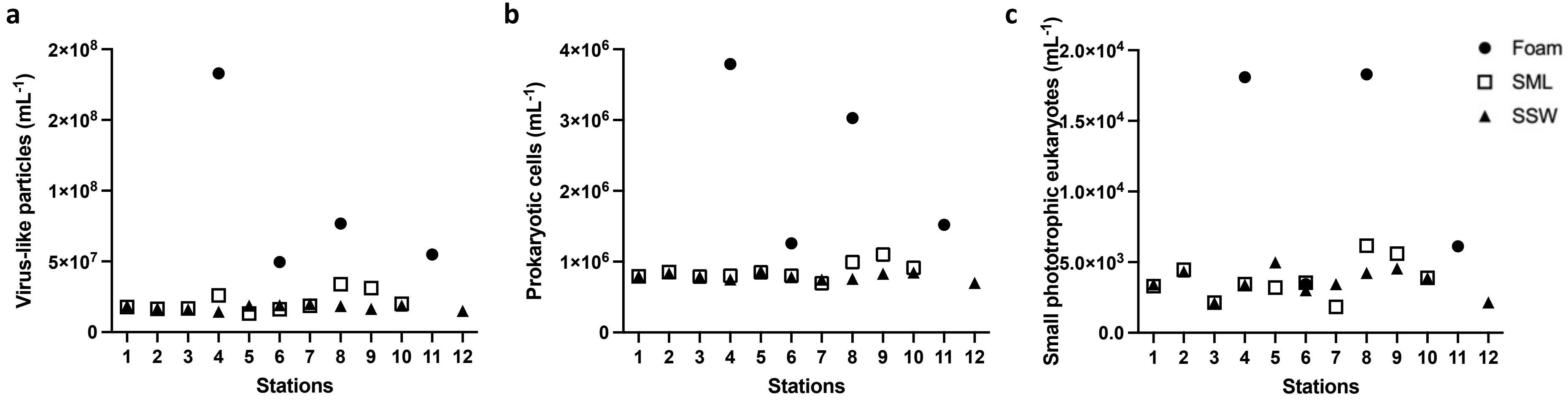

Supplementary Figure 1: Absolute counts of virus-like particles (VLP) (a), prokaryotic cells (b) and small phototrophic eukaryotes mL^{-1} (c) as counted in the flow cytometer across 12 sampled stations. SML= surface microlayer, SSW= subsurface water

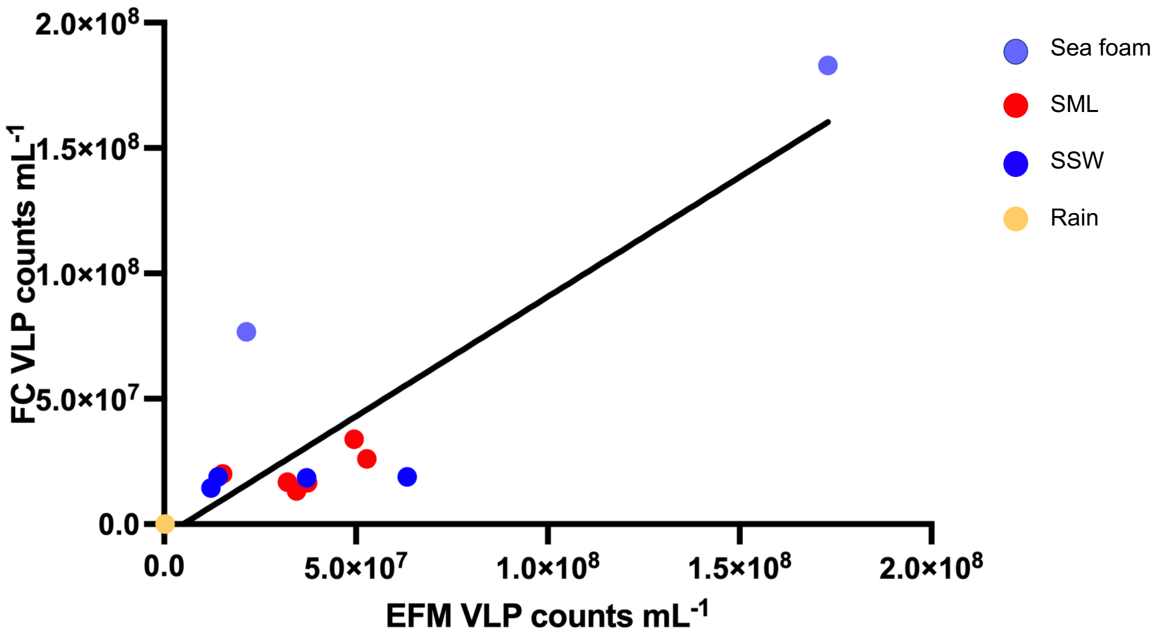

Supplementary Figure 2: Linear regression for virus-like particles (VLP) from rain (n = 1), foam (n = 2), surface microlayer (n = 5) and subsurface water (n = 5) counted in flow cytometry (FC) versus virus-like particles counted under the epifluorescence microscope (EFM). The highest value corresponds to a foam sample. Spearman rank correlation coefficient = 0.53.

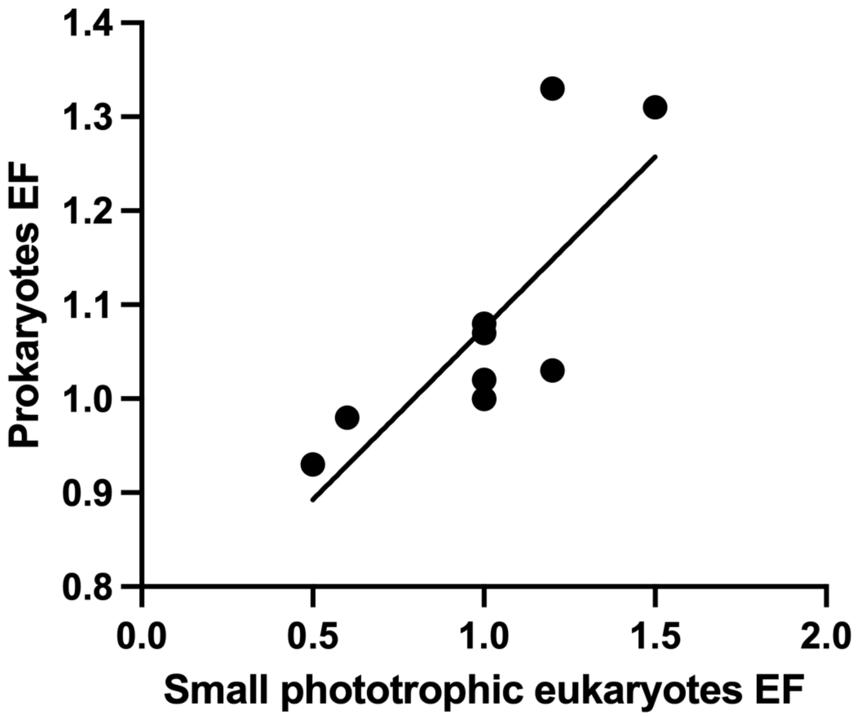

Supplementary Figure 3: Enrichment factors (EF) of prokaryotic cells versus EF of small phototrophic eukaryotes in the surface microlayer over subsurface water. Two-sided Spearman rank test indicates a significant ($p = 0.0039$, $n = 10$) correlation with Spearman's correlation coefficient = 0.8264 between both parameters.

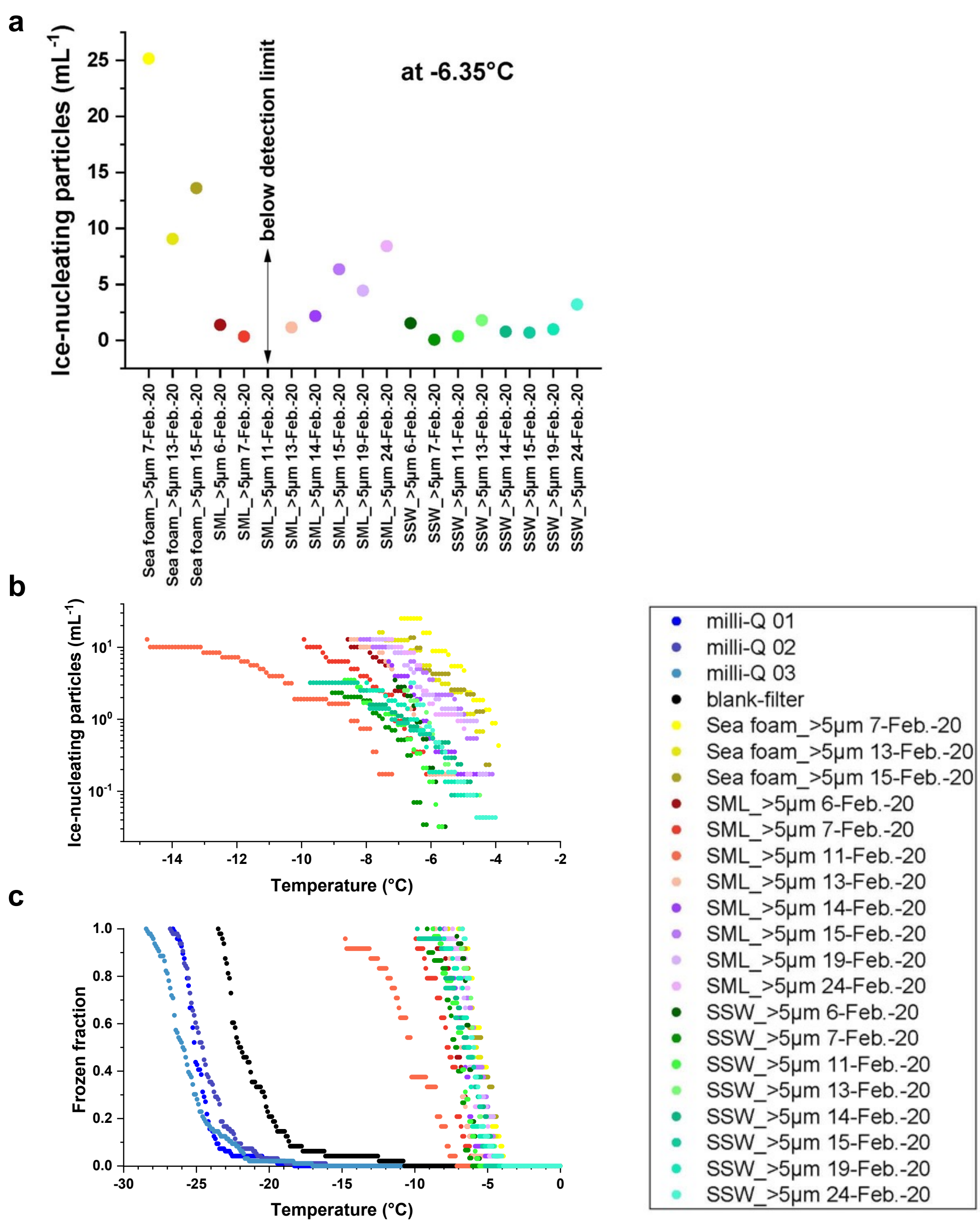

Supplementary Figure 4: Results from measurements of ice-nucleating particles (INP) in foams, surface microlayer (SML) and subsurface water (SSW). INP concentrations at the ice nucleation temperature of -6.35 °C (a), INP spectra over the detectable temperature range (b) and the measured frozen fraction values in comparison with results from pure water (c) showing that all samples were well above the background.

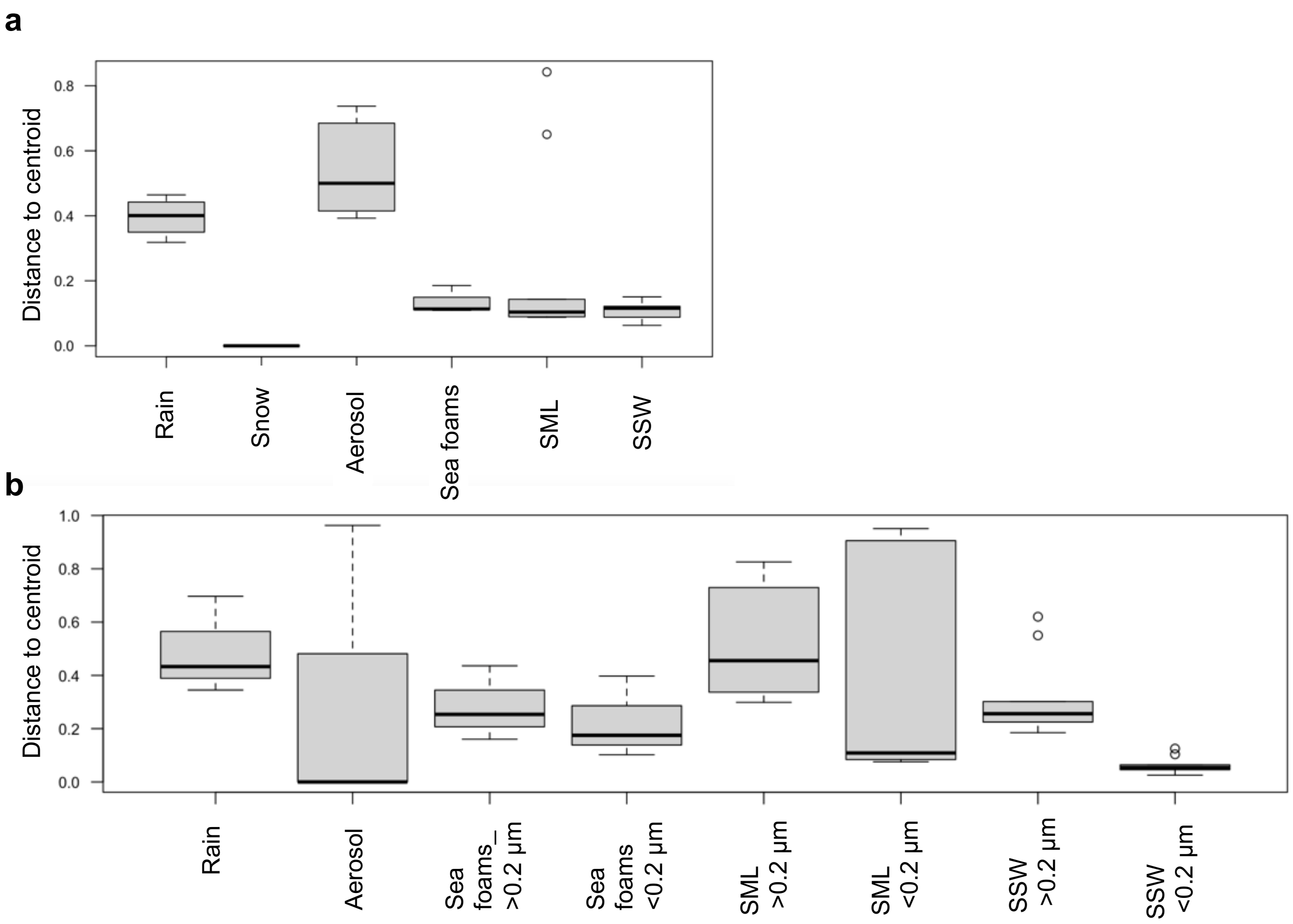

Supplementary Figure 5: Boxplots showing betadispers distances to centroid for various ecosystems and associated community of prokaryotes, with n = 4, 1, 8, 3, 9, 9 for the different samples in the order presented (a) and top 200 viruses with n = 3, 3, 3, 3, 9, 9, 9, 9 for the different samples in the order presented (b). Homogeneity of multivariate dispersions were calculated using the 'vegan' package in R and correspond to NMDS plots in Fig. 3b and Fig. 4b. The center line represents the median, box limits are upper and lower quartile, whiskers indicate highest and lowest values and dots are outliers. SML = surface microlayer, SSW = subsurface water

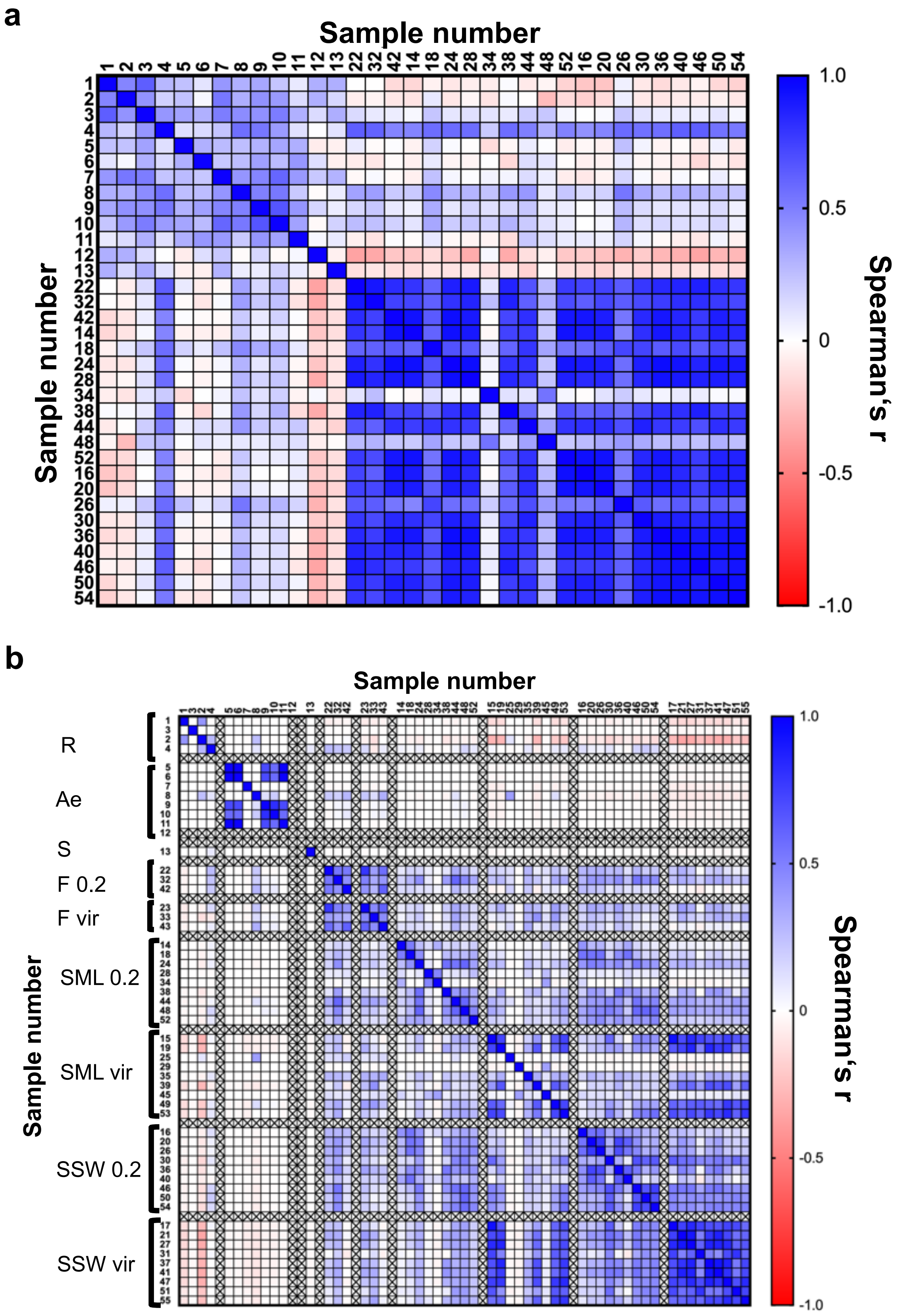

Supplementary Figure 6: Correlation matrix for relative abundances (based on read-normalized coverage) of prokaryotic taxa (*n* = 69) detected by rps3 gene prediction as shown in Figure 2 across rain, snow, aerosol, foam, surface microlayer (SML) and subsurface water (SSW) samples (a). Correlation matrix for abundances (based on read-sum normalized

coverage) of 1813 viral scaffolds across rain (R), snow (S), aerosol (Ae), foam (F), SML, and SSW samples (b). Sample numbers are in accordance with Supplementary data 12, vir = viromes, 0.2 = 0.2 µm fraction. Spearman *rho*, *p* values and confidence intervals for each pairwise comparison (two-sided test) shown in the correlation matrices are given in Supplementary data 13 & 14.

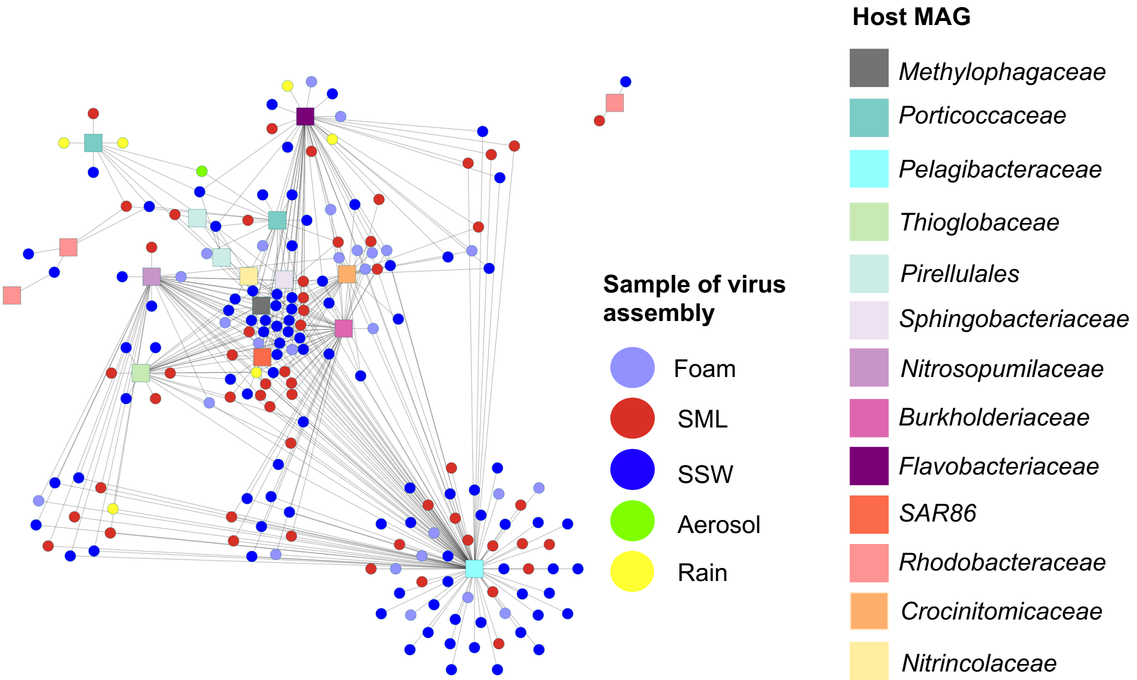

Supplementary Figure 7: Virus-host interaction based on k-mer frequency patterns. Using VirhostMatcher at a d2* dissimilarity threshold of < 0.3, viral scaffolds (represented as circles) were tested against a set of 26 dereplicated host MAGs (squares). Visualization was performed in Cytoscape v.9.3.

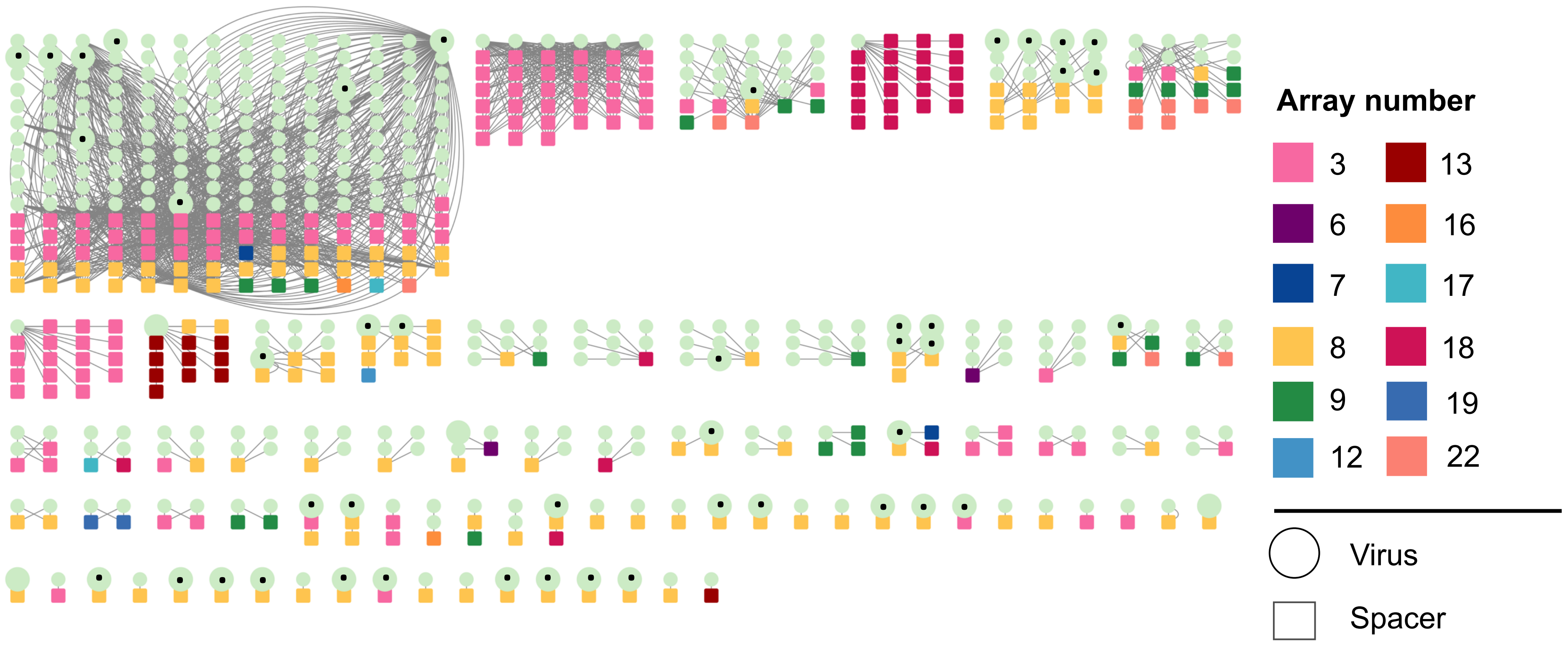

Supplementary Figure 8: Matches of CRISPR spacers to viral protospacers separated by CRISPR array. Squares represent spacers with different colors indicating different CRISPR array origins. Circles represent viruses with viruses assembled from rainwater being enlarged and viruses only detected in rain being further highlighted by a dot in the circle. Spacers from dominant arrays 3 and 8 match most viruses but also tend to match viruses from different ecosystems with array 3 and 8 providing spacers for marine and rainwater viruses, respectively.

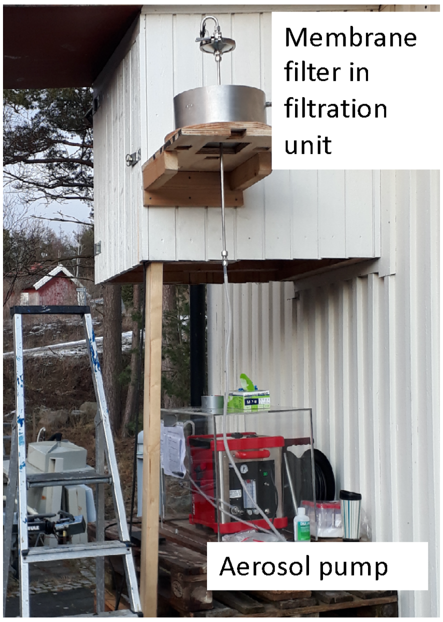

Supplementary Figure 9: Experimental set-up with aerosol pump and filter unit mounted at ~ 2 m above ground close to the coast.

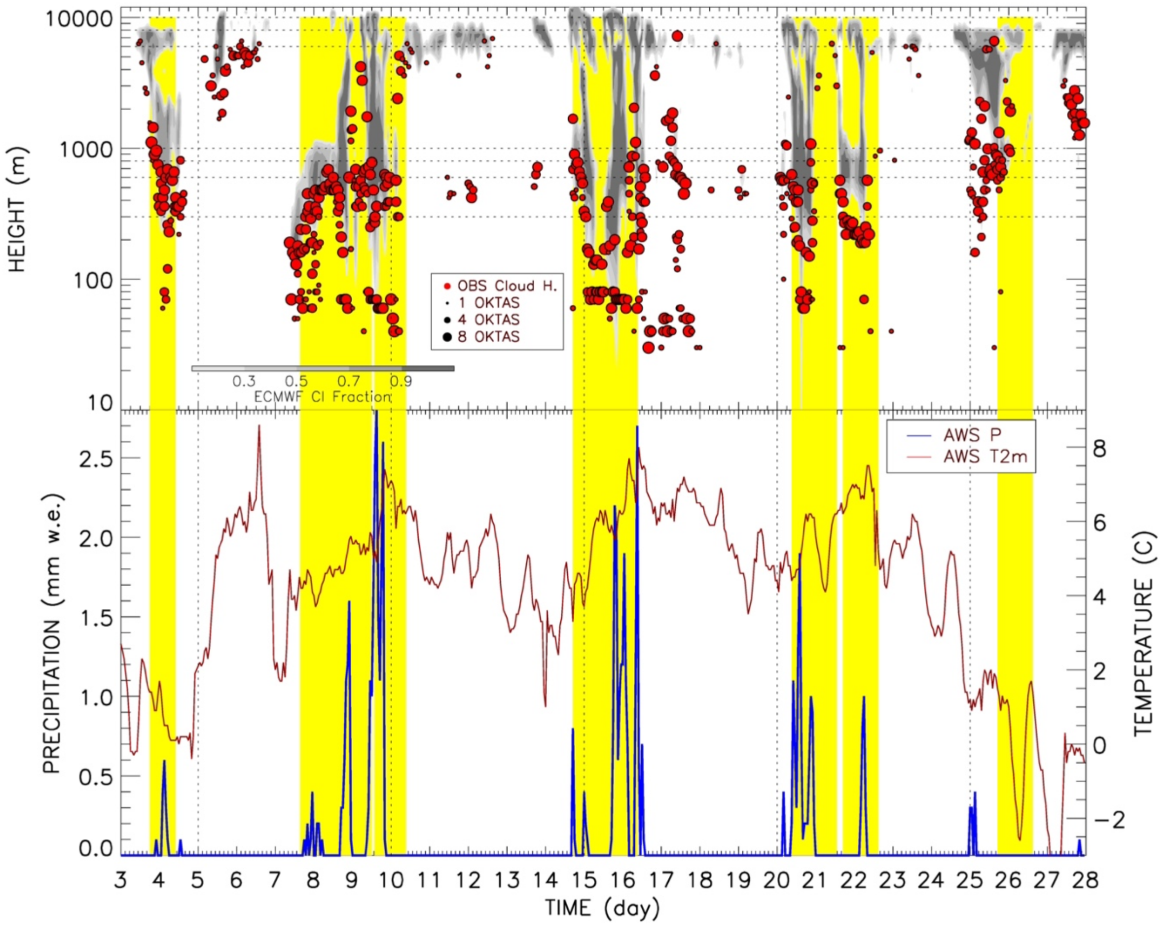

Supplementary Figure 10: Meteorological and cloud conditions at Nordkoster A from model and in situ measurements during February 2020. Time periods highlighted by yellow filled areas show rain sampling periods in both panels. Upper panel) cloud fraction (filled contour), and the cloud bases observed in situ (Red filled circles); the size of the circles shows the observed cloud coverage in OKTAS units (1 OKTAS=few clouds, 4 OCKTAS=scattered clouds, 8 OKTAS=overcast). Lower panel) Temperature at 2 meters above the ground (T2m) measured by the in situ Automatic Weather Station (AWS, red line). The blue line shows the precipitation quantity (P) in mm water equivalent (w.e.) measured by the AWS.

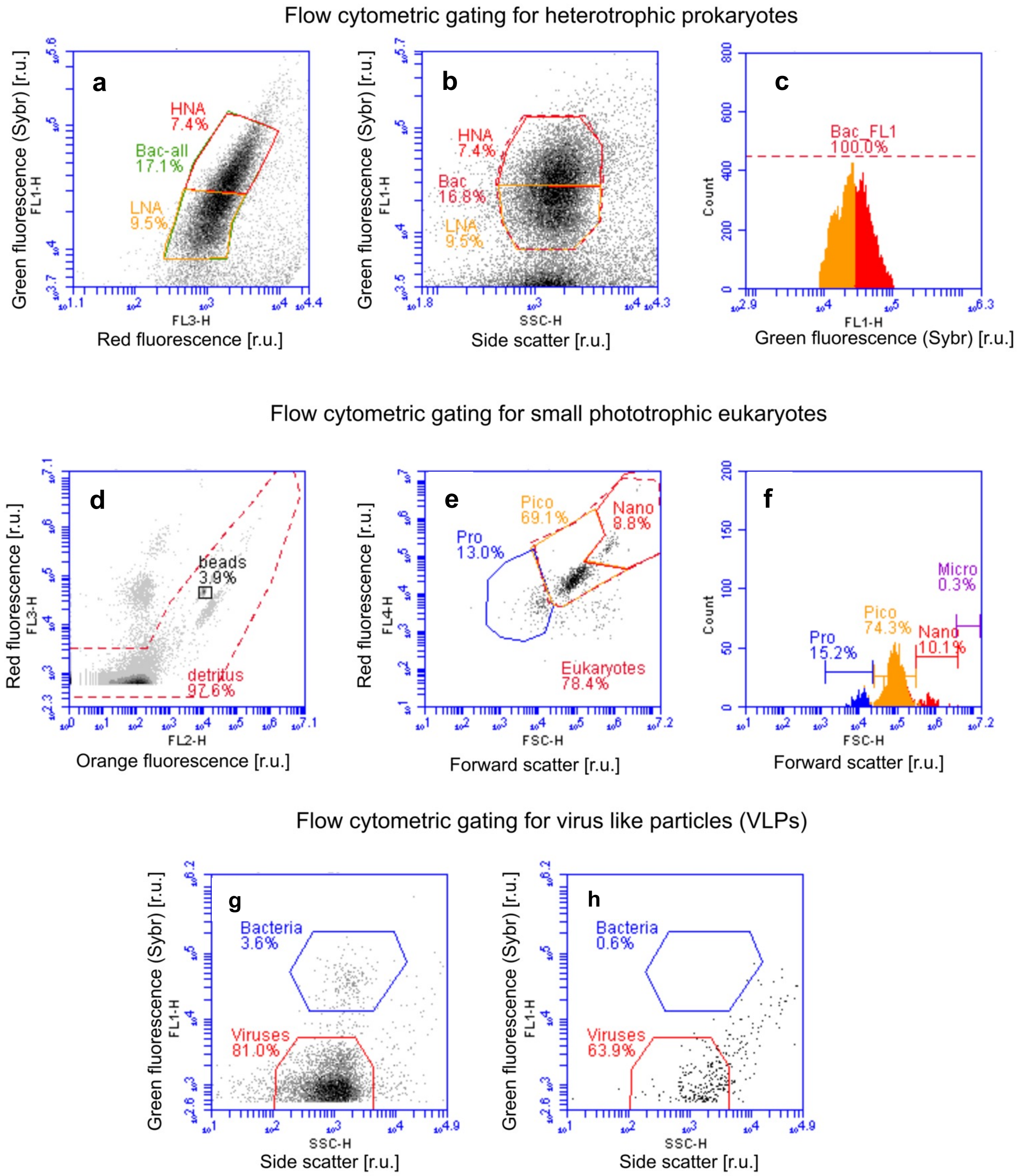

Supplementary Figure 11: Flow cytometric gating strategies for heterotrophic prokaryotes (a-c), small phototrophic eukaryotes (d-f), and virus-like particles (VLPs, g&h) using a BD Accuri C6 flow cytometer (BD Biosciences) with its Flow C software. Discrimination of phototrophic prokaryotes (a), double check of (sub-)populations against forward scatter (b) and their distribution as histogram (c). Discrimination of detritus, internal beads, and background noise for the autofluorescence of small phototrophic eukaryotes (d), regating of all events except detritus (e), histogram of the subpopulations of phototrophic pico-, nano-, and microplankton (f), gating of VLPs near detection limit of the device (threshold = 800). Potential subpopulations were not considered by extra gating (g) and determination of threshold noise of stained blank (sterile buffer (TE)), which was later subtracted from the VLP samples (h). Phototrophic prokaryotes were discriminated from the analysis due to the insufficient detection by the Accuri C6 after Ribeiro et al. (2016). r.u. = relative units.

References:

1. Ribeiro, C.G., Marie, D., dos Santos, A.L., Bandini, F.P., and Vaulot, D. Estimating microbial populations by flow cytometry: Comparison between instruments. *Limnol. Oceanogr. Methods* **14**, 750-758. doi: 10.1002/lom3.10135 (2016).
