# Supplementary material for: Marine viruses disperse bidirectionally along the natural water cycle
Source: Nat Commun. 2023 Oct 10;14:6354. doi: 10.1038/s41467-023-42125-5 (PMC10564846; doi:10.1038/s41467-023-42125-5)
Supplement: Supplementary file 3 — Description of Additional Supplementary Files [file 41467_2023_42125_MOESM3_ESM.pdf]

Description of Additional Supplementary Files

Title: Supplementary Movie 1

Description: The supplementary video shows extended sea foam patches floating at the air-sea interface in the Swedish Skagerrak.

Title: Supplementary data 1

Description: Metagenome assembled genomes (MAGs) with assigned taxonomy and genomic characteristics

Title: Supplementary data 2

Description: GTDB-tk output

Title: Supplementary data 3

Description: Proportion of metagenome-assembled genome covered with reads at coverage=1 across different samples and ecosystems; threshold for "presence" of MAG is 0.9 as indicated by green

Title: Supplementary data 4

Description: Information on viral clusters (VC) defined by vConTACT2. Level, weight and accession are parameters determined by graphanalyzer (https://github.com/lazzarigioele/graphanalyzer)

Title: Supplementary data 5

Description: Single nulceotide polymorphism (SNP) analysis for Virus_1 (see main Fig. 6) and associated mappings. SNP analysis performed in Geneious v11.1.5.

Title: Supplementary data 6

Description: Sampling times and volumes for air filtration; Sampled banks refer to handling filters as mentioned in the methods section of the main manuscript.

Title: Supplementary data 7

Description: Annotations for 109 viral scaffolds from rainwater provided by DRAM-v

Title: Supplementary data 8

Description: CRISPR arrays and consensus repeat sequence found on assemblies and MAGs using CRISPRcasFINDER (A), and spacer to protospacer hits after filtering at 80% similarity (B). In B, the array number is the first number behind "P" in the spacer name

Title: Supplementary data 9

Description: Field data for water sampling and notes, SML=surface microlayer, SSW=subsurface water

Title: Supplementary data 10

Description: Compiled information on precipitation samples, n.d.=not determined

Title: Supplementary data 11

Description: Accession numbers for metagenome-assembled genomes (MAGs) and raw data deposited in public databases

Title: Supplementary data 12

Description: Metagenome sample numbers

Title: Supplementary data 13

Description: Appendix of statistical data for Spearman rank correlation matrix of prokaryote data corresponding to Supplementary Figure 6

Title: Supplementary data 14

Description: Appendix of statistical data for Spearman rank correlation matrix of virus data corresponding to Supplementary Figure 6
