# Supplementary material for: Marine viruses disperse bidirectionally along the natural water cycle
Source: Nat Commun. 2023 Oct 10;14:6354. doi: 10.1038/s41467-023-42125-5 (PMC10564846; doi:10.1038/s41467-023-42125-5)
Supplement: Supplementary file 19 — Reporting Summary [file 41467_2023_42125_MOESM19_ESM.pdf]

nature portfolio

Corresponding author(s): Janina Rahlff

Last updated by author(s): 28.08.2023

Reporting Summary

Nature Portfolio wishes to improve the reproducibility of the work that we publish. This form provides structure for consistency and transparency in reporting. For further information on Nature Portfolio policies, see our Editorial Policies and the Editorial Policy Checklist.

Statistics

For all statistical analyses, confirm that the following items are present in the figure legend, table legend, main text, or Methods section.

n/a	Confirmed	
☐	☒	The exact sample size (*n*) for each experimental group/condition, given as a discrete number and unit of measurement
☐	☒	A statement on whether measurements were taken from distinct samples or whether the same sample was measured repeatedly
☐	☒	The statistical test(s) used AND whether they are one- or two-sided<br>*Only common tests should be described solely by name; describe more complex techniques in the Methods section.*
☐	☒	A description of all covariates tested
☐	☒	A description of any assumptions or corrections, such as tests of normality and adjustment for multiple comparisons
☐	☒	A full description of the statistical parameters including central tendency (e.g. means) or other basic estimates (e.g. regression coefficient) AND variation (e.g. standard deviation) or associated estimates of uncertainty (e.g. confidence intervals)
☐	☒	For null hypothesis testing, the test statistic (e.g. *F*, *t*, *r*) with confidence intervals, effect sizes, degrees of freedom and *P* value noted<br>*Give P values as exact values whenever suitable.*
☒	☐	For Bayesian analysis, information on the choice of priors and Markov chain Monte Carlo settings
☒	☐	For hierarchical and complex designs, identification of the appropriate level for tests and full reporting of outcomes
☒	☐	Estimates of effect sizes (e.g. Cohen's *d*, Pearson's *r*), indicating how they were calculated

Our web collection on statistics for biologists contains articles on many of the points above.

Software and code

Policy information about availability of computer code

Data collection	Light measurement in the field: Galaxy Sensors smartphone application v.1.8.10.<br>Epifluoresence microscopy software: EOS Utility version 2.10.2.0<br>Flow cytometry: BD Accuri Flow C Software, version 1.0.0264.21, build 20120423.264.21<br>Trajectory analysis: IDL (Interactive Data Language) software - version 8.7.2
Data analysis	Analysis of microscopic images: ImageJ, version 1.52<br>Statistics: R version 4.0.3 in R studio v.1.3.1093 including packages phyloseq 1.34.0, ggplot2 v.3.3.5., vegan v.2.5-7, GraphPad Prism v.9<br>Plotting of Graphs: R version 4.0.3 in R studio v.1.3.1093 (including packages phyloseq 1.34.0, ggplot2 v.3.3.5), GraphPad Prism v.9, OriginPro 2020 (64-bit) SR1 9.7.0.188 (Government)<br>Concept Figure: Adobe Express<br>Read QC: bbduk (https://github.com/BioInfoTools/BBMap/blob/master/sh/bbduk.sh) and Sickle<br>Subtraction of reads mapping to blank samples: https://github.com/ProbstLab/viromics/tree/master/extract_unmapped_stringent<br>Assemblies: MetaviralSPAdes v.3.14.0 and MetaSPAdes version 3.13 & 3.14<br>Viral genome identification: VIBRANT v.1.2.1., VirSorter v.1, ViralVerify v1.0<br>Viral clustering: VIRIDIC v.1.0<br>QC viral scaffolds: CheckV v.0.7.0 (database 0.6)<br>Gene prediction, annotation, clustering, taxonomic assignment: Prodigal v.2.6.3, DIAMOND v.0.88 blast, CD-HIT v.4.8.1 , USEARCH v.10.0.240_i86linux64<br>Virus functional annotations: DRAM-v v.1.2.4<br>SNP analysis: Geneious v.11.1.5

Synteny viral scaffolds: Easyfig v.2.2.5
Viral clustering and visualization: vCONTACT2 v.0.9.19 and Cytoscape v.3.9, with database version Dec.2021 from https://github.com/RyanCook94/inphared), compilation with graphanalyzer v.1.5.1 (https://github.com/lazzarigioele/graphanalyzer)
Virus identification (closest phylogenetic affiliation): PhaGCN2.0
Binning and curation: MetaBAT v.2.15 & Maxbin2 v.2.2.7, DasTool v.1.1.1, uBin v.0.9.14., dRep v.3.2.2
Bin QC: CheckM v.1.1.3
Bin taxonomy: GTDB-tk v.1.7.0 (database release r202)
Read mapping: Bowtie2 v.2.3.5.1
alpha and beta-diversity calculation: phyloseq 1.34.0 in R version 4.0.3.
CRISPR analysis: CRISPRCasFinder v. 4.2.20, MetaCRAST, BLASTn
Venn Diagrams: https://bioinformatics.psb.ugent.be/webtools/Venn/
Breadth calculation: https://github.com/ProbstLab/viromics/tree/master/calcopo
GC content calculation: https://github.com/ProbstLab/uBin-helperscripts/blob/master/bin/04_02gc_count.rb
Coverage calculation: https://github.com/ProbstLab/uBin-helperscripts/blob/master/bin/04_01calc_coverage_v3.rb
k-mer linking viruses to MAGs: VirusHostMatcher v.1.0.0
General analyses: Excel, Microsoft Office Professional Plus 2016; Microsoft Excel for Mac v.16.62

For manuscripts utilizing custom algorithms or software that are central to the research but not yet described in published literature, software must be made available to editors and reviewers. We strongly encourage code deposition in a community repository (e.g. GitHub). See the Nature Portfolio guidelines for submitting code & software for further information.

Data

Policy information about availability of data

All manuscripts must include a data availability statement. This statement should provide the following information, where applicable:

- Accession codes, unique identifiers, or web links for publicly available datasets
- A description of any restrictions on data availability
- For clinical datasets or third party data, please ensure that the statement adheres to our policy

ECMWF ERA5 reanalysis data are freely available on Copernicus Climate Change Service (C3S) Climate Data Store (CDS) under: https://doi.org/10.24381/cds.bd0915c6 and https://doi.org/10.24381/cds.adbb2d47. Flow cytometry data have been stored at PANGAEA database (https://doi.pangaea.de/10.1594/PANGAEA.947348) and linked to the Integrated Marine Information System (IMIS, https://www.vliz.be/en/imis?module=dataset&dasid=8313). Epifluorescence microscopy pictures (https://figshare.com/articles/figure/Epifluorescence_microscopy_images_of_virus-like_particle_from_rainwater_sea_foam_surface_microlayer_and_1_m_deep_water_rainwater_of_the_Swedish_Skagerrak/22002116) , trajectories (https://figshare.com/articles/dataset/Trajectories/23808678), and the viral network (https://figshare.com/articles/dataset/c1_ntw_vConTACT_files_virome_network/23807859) are available at figshare. The Viral Refseq database (release December 2021) is available from INPHARED (https://github.com/RyanCook94/inphared/tree/5293f5474a65743a357e6b94a3660a0adbf33580). All sequencing data, MAGs, and the viral metagenome are stored in Bioproject PRJNA811790 (https://www.ncbi.nlm.nih.gov/bioproject/PRJNA811790). For further details on accession numbers, please refer to Supplementary data 11. Source data are provided with this paper.

Research involving human participants, their data, or biological material

Policy information about studies with human participants or human data. See also policy information about sex, gender (identity/presentation), and sexual orientation and race, ethnicity and racism.

Reporting on sex and gender	n/a
Reporting on race, ethnicity, or other socially relevant groupings	n/a
Population characteristics	n/a
Recruitment	n/a
Ethics oversight	n/a

Note that full information on the approval of the study protocol must also be provided in the manuscript.

Field-specific reporting

Please select the one below that is the best fit for your research. If you are not sure, read the appropriate sections before making your selection.

☐ Life sciences ☐ Behavioural & social sciences ☒ Ecological, evolutionary & environmental sciences

For a reference copy of the document with all sections, see nature.com/documents/nr-reporting-summary-flat.pdf

Ecological, evolutionary & environmental sciences study design

All studies must disclose on these points even when the disclosure is negative.

Study description

Samples from the air-sea boundary (1m deep water, surface microlayer and sea foams), aerosols and rainwater were sampled in a coastal region in the Skagerrak, Tjärnö, Sweden during one month in February 2020. Metagenome samples as shown in Supplementary Table 11 were sequenced. Flow cytometric analyses of cell and virus particle counts were performed for seawater (n =11), microlayer (n=10), foam (n = 3), and rainwater samples (n = 4). Since sampling of microlayer and foam is highly dependent on weather conditions such as wind speed, we could not plan beforehand the number of days that sampling is possible. A single sample of each feature (foams, SML etc.) was collected per station. Duplicate samples were taken for flow cytometry. Concentration of ice-nucleating particles and trajectories for air masses related to rain events were calculated.

Research sample

The metagenome samples represent natural microbial and viral communities from the environment (source: boundary layer aerosols, foams, microlayer, seawater, rainwater). We aimed to study cross-ecosystem dispersal of viruses from the air-water boundary via aerosols to rain. After a pre-filtration on 5 µm pore size filters, seawater and rain samples were filtered on 0.2 µm pore size (representing the microbial community and absorbed viruses), and the flow-through was chemically flocculated and represented the viral fraction. The microbial and viral fraction were sequenced for metagenomics and viromics.

Sampling strategy

Sample size was chosen according to arising opportunities (in case of rainwater) and in dependence on weather conditions (in case of air-sea boundary sampling), as high wind speeds prevent sampling the sea surface with the glass plate. Aerosol filters were changed every 24-96 hours, due to uncertainty about the best sampling duration (trade-off between sufficient DNA yield and sampling resolution). Microlayer and foams were sampled with the glass plate method, as this is one of the state-of-the-art methods for microbiological sampling.

Data collection

The authors Janina Rahlff and Sarah P. Esser conducted the sampling. For sampling, the engine of the boat was switched off. JR and SPE wore nitril gloves during the sampling. At sea, JR submerged the pre-rinsed glass plate into the water and SPE wiped-off the sample in a clean bottle. SPE collected the 1 m deep water. Wind speed, position, light conditions, water temperature and salinity were typically recorded before the sampling and written into a waterproof journal.

Timing and spatial scale

Seawater sampling sites were located in the bay offshore Tjärnö, Swedish west coast in the Skagerrak. The position, time, and dates for the samplings are given below. Sea surface sampling with the small boat was dependent on low wind speeds and sometimes prevented by stormy conditions. We performed it as often as possible. Foam sampling cannot be planned, we sampled foams anytime we found some. Aerosols were sampled continuously throughout the 1-month period and rainwater whenever it fell.

Supplementary data 9: Field data for water sampling and notes, SML=surface microlayer=first 1mm, SSW=subsurface water=1m depth
Station; Latitude; Longitude; Date; Wind speed at sea (m s-1); Water Salinity; Water temp. (°C); Light (Lux); Time of water sampling (local); Sample collected; Weather conditions/notes
1; 58.8801125; 11.1071915; 03.02.20; 1; 25.4; 4.2; 7330; 13:30-14:45; SML, SSW; sunny, calm sea
2; 58.880872; 11.1112471; 04.02.20; 3.4; 28.8; 4.5; 2850; 9:15-10:15; SML, SSW; cloudy, snow in the night before, surface looks like a slick
3; 58.881814 11.110815; 06.02.20; 1; 25.9; 4.7; 7413; 14:30-15:30; SML, SSW; sunny, some clouds, calm surface, starting sampling in a slick
4; 58.883268; 11.105827; 07.02.20; 3; 27.1; 4; 3078; 9:00-10:00; Foam, SML, SSW; no clouds, very cold (0 degree), sun in the beginning
5; 58.881934; 11.12074; 11.02.20; 6; 30; 5.4; 6010; 13:48-14:30; SML, SSW; sunny, some clouds, beginning white cap formation, water well mixed/turbid
6; 58.88082; 11.115091; 13.02.20; 2.6 - 5.0; 24.4; 4.5; 4750; 13:20-14:30; Foam, SML, SSW; sunny, some clouds
7; 58.876092; 11.110915; 14.02.20; 0; 22.5; 3.3; 2540; 9:25-10:14; SML, SSW; sunny, little wind, surface like a mirror, clear water
8; 58.88679; 11.106764; 15.02.20; 4.8; 27.2; 4.7; 2517; 14:45-15:30; Foam, SML, SSW; tight cloud cover, small waves w/o white caps, grey weather like it could starting raining
9; 58.885847; 11.106157; 19.02.20; 3.1; 27.8; 4.8; 5011; 10:17-10:45; SML, SSW; sunny, blue sky, no clouds, some waves w/o white caps
10a; 58.880987; 11.136508; 24.02.20; 4.4-5.6; 27; 3.5; 7520; 13:31-15:10; SML, SSW; sun, some clouds, beginning white caps, sea flattened and wind speed decreased during sampling
10b; 58.879518; 11.124977; 24.02.20; 4.4-5.6; 27; 3.5; 7520; 13:31-15:10; Foam;
11; 58.876299; 11.109517; 26.02.20; 5.2-6.0; 28.8; 4.3; 6120; 13:35-13:45; SSW; sunny, few clouds, wavy w/o white caps, SSW sampling for flow cytometry only
12/R; 58.8765692; 11.1463651; permanent station for aerosol and precipitation sampling

Aerosol sampling table (Supplementary data 6)
Sampling_date; Aerosol Vol norm. (m3); Aerosol duration of filtering (h); Blank sampled; Notes
13.-15. Feb. 20; 36.4; 50; yes; some rain on filter
06.-07. Feb. 20; 19.0; 24; no
07.-10. Feb. 20; 46.7; 75; yes; some rain on filter
10.-13. Feb. 20; 51.1; 72; no;
15.-19. Feb. 20; 61.0; 96; no; some rain on filter
19.-23. Feb. 20; 56.6; 96.5; yes; some rain on filter
23.-25. Feb. 20; 31.7; 47.5; no; some rain on filter
25.-27. Feb. 20; 32.6; 46; yes;

Rain sampling (see Supplementary data 10):
Event; Date and Time;
Event 1: 07.Feb.20 15:30 - 09.02.20 12:00
Event 2: 14.Feb.20 17:00 - 16.02.20 9:15
20.Feb.20 9:00 - 21.02.20 13:00
21.Feb.20 17:00 - 22.Feb.20 15:00 (pool of 3 DNA samples)

Data exclusions

Rain samples not included in this study were excluded due to low DNA yield. Some rain samples had to be pooled to gain enough DNA for sequencing as stated in the Methods section.

Reproducibility

Our sampling strategy involved repeated water and continuous aerosol sampling over a 1-month period in the same coastal area. Water samples were retrieved from eleven stations at eleven different days. Rain events cannot be planned or reproduced and were sampled as they have happened. For rain samples please refer to Supplementary data 10.

Randomization

Marine environments are naturally diverse and can have significant spatial variation in water properties, such as temperature, salinity, nutrient levels, and biological communities. Water properties also naturally vary over time due to tides, currents, seasonal changes, and other natural processes. By sampling on different days, temporal-spatial variability of water masses occurs naturally and allows to gain insights into how the marine environment changes without extra randomization. The sampling position was additionally changed every day.

Blinding

Blinding involves withholding certain information from participants or researchers to prevent preconceived notions or expectations from influencing the study outcomes. Our study did not involve human or animal study participants or the need to withhold information from them to influence our study's outcome.

Did the study involve field work? ☒ Yes ☐ No

Field work, collection and transport

Field conditions

Field conditions for 12 stations are listed in Supplementary data 9, as follows
Supplementary data 9: Field data for water sampling and notes, SML=surface microlayer=first 1mm, SSW=subsurface water=1m depth
Station; Latitude; Longitude; Date; Wind speed at sea (m s-1); Water Salinity; Water temp. (°C); Light (Lux); Time of water sampling (local); Sample collected; Weather conditions/notes
1; 58.8801125; 11.1071915; 03.02.20; 1; 25.4; 4.2; 7330; 13:30-14:45; SML, SSW; sunny, calm sea
2; 58.880872; 11.1112471; 04.02.20; 3.4; 28.8; 4.5; 2850; 9:15-10:15; SML, SSW; cloudy, snow in the night before, surface looks like a slick
3; 58.881814 11.110815; 06.02.20; 1; 25.9; 4.7; 7413; 14:30-15:30; SML, SSW; sunny, some clouds, calm surface, starting sampling in a slick
4; 58.883268; 11.105827; 07.02.20; 3; 27.1; 4; 3078; 9:00-10:00; Foam, SML, SSW; no clouds, very cold (0 degree), sun in the beginning
5; 58.881934; 11.12074; 11.02.20; 6; 30; 5.4; 6010; 13:48-14:30; SML, SSW; sunny, some clouds, beginning white cap formation, water well mixed/turbid
6; 58.88082; 11.115091; 13.02.20; 2.6 - 5.0; 24.4; 4.5; 4750; 13:20-14:30; Foam, SML, SSW; sunny, some clouds
7; 58.876092; 11.110915; 14.02.20; 0; 22.5; 3.3; 2540; 9:25-10:14; SML, SSW; sunny, little wind, surface like a mirror, clear water
8; 58.88679; 11.106764; 15.02.20; 4.8; 27.2; 4.7; 2517; 14:45-15:30; Foam, SML, SSW; tight cloud cover, small waves w/o white caps, grey weather like it could starting raining
9; 58.885847; 11.106157; 19.02.20; 3.1; 27.8; 4.8; 5011; 10:17-10:45; SML, SSW; sunny, blue sky, no clouds, some waves w/o white caps
10a; 58.880987; 11.136508; 24.02.20; 4.4-5.6; 27; 3.5; 7520; 13:31-15:10; SML, SSW; sun, some clouds, beginning white caps, sea flattened and wind speed decreased during sampling
10b; 58.879518; 11.124977; 24.02.20; 4.4-5.6; 27; 3.5; 7520; 13:31-15:10; Foam;
11; 58.876299; 11.109517; 26.02.20; 5.2-6.0; 28.8; 4.3; 6120; 13:35-13:45; SSW; sunny, few clouds, wavy w/o white caps, SSW sampling for flow cytometry only
12/R; 58.8765692; 11.1463651; permanent station for aerosol and precipitation sampling

Aerosol sampling table (Supplementary data 6)
Sampling_date; Aerosol Vol norm. (m3); Aerosol duration of filtering (h); Blank sampled; Notes
13.-15. Feb. 20; 36.4; 50; yes; some rain on filter
06.-07. Feb. 20; 19.0; 24; no
07.-10. Feb. 20; 46.7; 75; yes; some rain on filter
10.-13. Feb. 20; 51.1; 72; no;
15.-19. Feb. 20; 61.0; 96; no; some rain on filter
19.-23. Feb. 20; 56.6; 96.5; yes; some rain on filter
23.-25. Feb. 20; 31.7; 47.5; no; some rain on filter
25.-27. Feb. 20; 32.6; 46; yes;

Rain sampling (see Supplementary data 10):
Event; Date and Time;

	Event 1: 07.Feb.20 15:30 - 09.02.20 12:00<br>Event 2: 14.Feb.20 17:00 - 16.02.20 9:15<br>20.Feb.20 9:00 - 21.02.20 13:00<br>21.Feb.20 17:00 - 22.Feb.20 15:00 (pool of 3 DNA samples)
Location	Seawater sampling sites were located in the bay offshore Tjärnö, Swedish west coast in the Skagerrak. The position, time, and dates for the samplings are given below.<br>Supplementary data 9: Field data for water sampling and notes, SML=surface microlayer=first 1mm, SSW=subsurface water=1m depth<br>Station; Latitude; Longitude; Date; Wind speed at sea (m s-1); Water Salinity; Water temp. (°C); Light (Lux); Time of water sampling (local); Sample collected; Weather conditions/notes<br>1; 58.8801125; 11.1071915; 03.02.20; 1; 25.4; 4.2; 7330; 13:30-14:45; SML, SSW; sunny, calm sea<br>2; 58.880872; 11.1112471; 04.02.20; 3.4; 28.8; 4.5; 2850; 9:15-10:15; SML, SSW; cloudy, snow in the night before, surface looks like a slick<br>3; 58.881814 11.110815; 06.02.20; 1; 25.9; 4.7; 7413; 14:30-15:30; SML, SSW; sunny, some clouds, calm surface, starting sampling in a slick<br>4; 58.883268; 11.105827; 07.02.20; 3; 27.1; 4; 3078; 9:00-10:00; Foam, SML, SSW; no clouds, very cold (0 degree), sun in the beginning<br>5; 58.881934; 11.12074; 11.02.20; 6; 30; 5.4; 6010; 13:48-14:30; SML, SSW; sunny, some clouds, beginning white cap formation, water well mixed/turbid<br>6; 58.88082; 11.115091; 13.02.20; 2.6 - 5.0; 24.4; 4.5; 4750; 13:20-14:30; Foam, SML, SSW; sunny, some clouds<br>7; 58.876092; 11.110915; 14.02.20; 0; 22.5; 3.3; 2540; 9:25-10:14; SML, SSW; sunny, little wind, surface like a mirror, clear water<br>8; 58.88679; 11.106764; 15.02.20; 4.8; 27.2; 4.7; 2517; 14:45-15:30; Foam, SML, SSW; tight cloud cover, small waves w/o white caps, grey weather like it could starting raining<br>9; 58.885847; 11.106157; 19.02.20; 3.1; 27.8; 4.8; 5011; 10:17-10:45; SML, SSW; sunny, blue sky, no clouds, some waves w/o white caps<br>10a; 58.880987; 11.136508; 24.02.20; 4.4-5.6; 27; 3.5; 7520; 13:31-15:10; SML, SSW; sun, some clouds, beginning white caps, sea flattened and wind speed decreased during sampling<br>10b; 58.879518; 11.124977; 24.02.20; 4.4-5.6; 27; 3.5; 7520; 13:31-15:10; Foam;<br>11; 58.876299; 11.109517; 26.02.20; 5.2-6.0; 28.8; 4.3; 6120; 13:35-13:45; SSW; sunny, few clouds, wavy w/o white caps, SSW sampling for flow cytometry only<br>12/R; 58.8765692; 11.1463651; permanent station for aerosol and precipitation sampling
Access & import/export	Our samples were transported on dry ice from Sweden to Germany and there stored at -80°C. A member of the "Species Unit" of the Swedish Environmental Protection Agency confirmed that we would not need special sampling permission to comply with the Nagoya Protocol.
Disturbance	no disturbances identified

Reporting for specific materials, systems and methods

We require information from authors about some types of materials, experimental systems and methods used in many studies. Here, indicate whether each material, system or method listed is relevant to your study. If you are not sure if a list item applies to your research, read the appropriate section before selecting a response.

Materials & experimental systems

n/a	Involved in the study
☒	☐ Antibodies
☒	☐ Eukaryotic cell lines
☒	☐ Palaeontology and archaeology
☒	☐ Animals and other organisms
☒	☐ Clinical data
☒	☐ Dual use research of concern
☒	☐ Plants

Methods

n/a	Involved in the study
☒	☐ ChIP-seq
☐	☒ Flow cytometry
☒	☐ MRI-based neuroimaging

Flow Cytometry

Plots

Confirm that:

☒ The axis labels state the marker and fluorochrome used (e.g. CD4-FITC).

☒ The axis scales are clearly visible. Include numbers along axes only for bottom left plot of group (a 'group' is an analysis of identical markers).

☒ All plots are contour plots with outliers or pseudocolor plots.

☒ A numerical value for number of cells or percentage (with statistics) is provided.

Methodology

Sample preparation	Duplicates of unfiltered seawater, foam and precipitation samples were fixed with glutardialdehyde (1% final concentration, Merck, Sweden), stored for 1 hour in the dark and subsequently stored at -80°C until flow cytrometric analysis. Particle-enriched foams were gravity filtered onto 50 µm filters (CellTrics®, Sysmex Partec, Muenster, Germany) before cell counts determination of prokaryotes and small phototrophic (autofluorescent) eukaryotes.<br><br>Prokaryotic cell numbers were determined after the protocol of Giebel et al. (2019): In brief, the in an ice-bath thawed sample was stained by the DNA dye SybrGreen I (10X final conc., Invitrogen, The Netherlands). As internal standard and for performance monitoring, 1 µm multifluorescent latex beads (catalog no. 24062; Polysciences Europe, Germany) were used. After 30 min of incubation in the dark, each sample was analyzed for 2 min using a flow rate of 14 µl min−1. Samples with an event rate >1500 events s−1 were diluted with sterile seawater to avoid coincidence.<br>Giebel, H.A., Wolterink, M., Brinkhoff, T., Simon, M. Complementary energy acquisition via aerobic anoxygenic photosynthesis and carbon monoxide oxidation by Planktomarina temperata of the Roseobacter group. FEMS Microbiol Ecol 95, fiz050, doi:10.1093/femsec/fiz050 (2019).<br><br>Small eukaryotic phototrophic cell numbers were determined after the protocol of Giebel et al. (2021) and Marie et al. (2000): Slow thawed (ice bath) and unstained sample was mixed with internal standard beads and subsequently analyzed for 3 to 4 min using a flow rate of 66 µl min−1.<br>Giebel, H.A., Arnosti, C., Badewien, T.H., Bakenhus, I., Balmonte, J.P., Billerbeck, S., Dlugosch, L., Henkel, R., Kuerzel, B., Meyerjürgens, J., Milke, F., Voss, D., Wienhausen, G., Wietz, M., Winkler, H., Wolterink, M., Simon, M. Microbial Growth and Organic Matter Cycling in the Pacific Ocean Along a Latitudinal Transect Between Subarctic and Subantarctic Waters. Front. Mar. Sci. 8:764383, doi:10.3389/fmars.2021.764383 (2021)<br>Marie, D., Simon, N., Guillou, L., Partensky, F. & Vaulot, D. in In Living Color. Springer Lab Manuals (eds R.A. Diamond & S. Demaggio) Ch. Chapter 34, 421-454 (Springer, 2000).<br><br>Virus like particles (VLPs) were determined following exactly the protocol of Brussaard et al. (2010) using fixed samples with glutardialdehyde (final conc. 1%).<br>Brussaard, C. P. D. et al. Manual of aquatic viral ecology. Am Soc Limnol Oceanogr, 102-109 (2010).
Instrument	BD Accuri C6 (Beckton Dickinson Biosciences, Franklin Lakes, USA)
Software	BD Accuri Flow C Software, version 1.0.0264.21, build 20120423.264.21
Cell population abundance	Cell population abundance was determined after gating the distinct populations. Cell sorting was not applied to check for purity, because the C6 is not a cell sorter. Due to combining scatter characteristics with fluorescent properties, populations consists of the distinct organisms.
Gating strategy	Prokaryotes: Prokaryotes were detected using manual gating after visual inspection of the dot plots of the green (FL1, 530 ± 15 nm) versus red fluorescence (FL3, >670 nm) and forward versus sideward scatters (FSC and SSC) and their histogram plots, respectively. The threshold of the FL1 channel was set at 900.<br>VLPs: Population of VLPs is located next to the threshold of the green fluorescence (550 on FL1) vs. the sideward scatter (SSC) and the forward scatter signal, respectively. Potential subpopulations were not taken into account.<br>Small eukaryotic phototrophic cells: Autofluorescence of phototrophic eukaryotic cells was assessed after excitation by blue as well as red laser light (488 nm and 630 nm, respectively). Cell counts of autofluorescent pico-, nano- and microplankton were determined by re-gating data from a plot of fluorescence FL3 (red, >670 nm) vs. FL2 (orange, 585±20 nm) to a cytogram of FL4 (red, 675±12.5 nm) vs. forward scatter light (FSC). The gating strategy is depicted in Supplementary Figure 11.

☒ Tick this box to confirm that a figure exemplifying the gating strategy is provided in the Supplementary Information.
